# Urinary Tract Infection in Children: A Narrative Review

**DOI:** 10.7759/cureus.51469

**Published:** 2024-01-01

**Authors:** Priyansi Agrawal, Vaishali M Paunikar

**Affiliations:** 1 Medicine, Jawaharlal Nehru Medical College, Datta Meghe Institute of Higher Education and Research, Wardha, IND; 2 Physiology, Jawaharlal Nehru Medical College, Datta Meghe Institute of Higher Education and Research, Wardha, IND

**Keywords:** antibiotics, risk factors, pathophysiology, treatment, etiology, methods, diagnosis, urinary tract infection in children

## Abstract

This study investigates the susceptibility of different age groups and genders to urinary tract infections (UTIs) in pediatric populations, focusing on anatomical and behavioural factors. A systematic review of clinical data explores manifestations, accurate diagnosis methods, and antibiotic treatment regimens. Escherichia coli is a prevalent uropathogen, and the study addresses concerns about antibiotic resistance. The research aims to close knowledge gaps, influence guidelines, and enhance public health initiatives. Factors influencing UTI prevalence, such as age, gender, and structural abnormalities, are discussed. The review follows a robust search methodology, presenting a Preferred Reporting Items for Systematic Reviews and Meta-Analyses (PRISMA) flow diagram. The included studies cover a wide range of topics related to pediatric UTIs, including aetiology, treatment, prevention, and diagnostic approaches. The study emphasizes the importance of understanding and addressing pediatric UTIs for effective management and prevention.

## Introduction and background

This study aims to investigate the susceptibility of different age groups and genders to urinary tract infections (UTIs) by exploring anatomical and behavioural factors [1]. Through a systematic review of clinical data, the research will document a spectrum of clinical manifestations, ranging from nonspecific symptoms in infants to more definite symptoms in older children, with a focus on accurate diagnosis using urine culture and imaging methods, especially in febrile UTIs [2,3]. The predominant uropathogenic cause in pediatric UTIs is Escherichia coli, with infants often presenting with vague signs, including an unexplained fever [1,4]. The study focuses on antibiotic treatment regimens in light of the growing concern about antibiotic-resistant bacteria and potential long-term consequences [5,6]. Prophylactic antibiotics and their implications on pediatric health, such as the risk of renal scarring and subsequent hypertension, will also be investigated using a multidisciplinary approach [7].

Recognizing UTIs as common and severe bacterial infections in paediatrics, the study aims to address controversies in diagnosis and treatment, ultimately closing knowledge gaps and influencing professional guidelines, advice, and public health initiatives on pediatric UTIs [2,3]. The research explores the role of uropathogens and their modes of transmission, examining the link between juvenile UTIs and bladder dysfunctions as potential indicators of congenital kidney and urinary tract anomalies [4]. The prevalence of UTIs is influenced by age, gender, and various risk factors, with infants and young children, especially those under two, being more susceptible due to anatomical features [6]. Structural abnormalities contribute to UTIs in both males and females, with uncircumcised boys having a higher incidence in the first year of life and females of school age being more vulnerable due to their anatomical structure [7]. Conditions such as vesicoureteral reflux, obstructive uropathies, and urinary tract abnormalities also contribute to UTIs [7,8]. Various risk factors, including kidney stones, diabetes, sexual activity, and hereditary factors, play a role in older children and adolescents [9]. Recurrent UTIs are noted, particularly in children with underlying medical issues [10].

## Review

Search methodology

**Figure 1:**
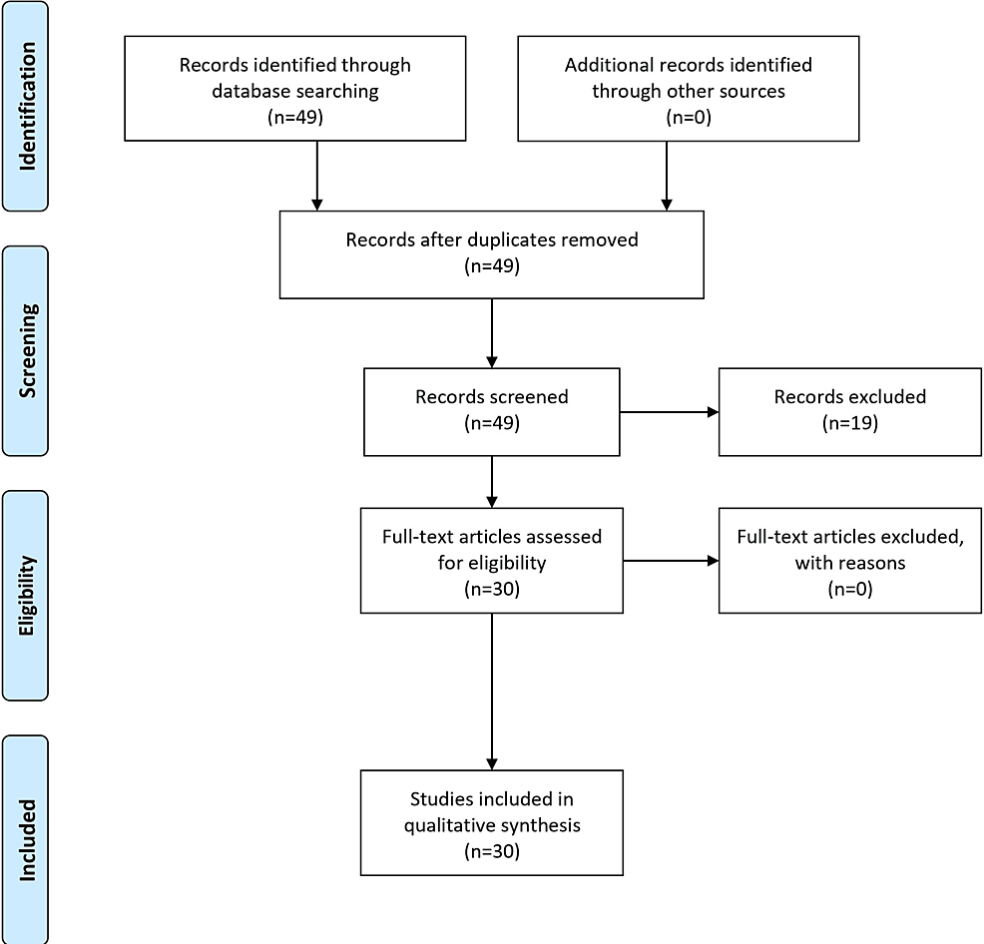
PRISMA flow diagram PRISMA: Preferred Reporting Items for Systematic Reviews and Meta-Analyses

The goal of the research article on pediatric UTIs is to locate and evaluate studies that address the causes, methods of diagnosis, and treatment of pediatric UTIs. We used electronic resources such as ClinicalTrials.gov, Embase (Excerpta Medica Database), Google Scholar, Cochrane Library, and PubMed for our search. With "Pediatric" OR "Children" AND ("Urinary Tract Infections" OR "UTIs") AND a few more related phrases, our search string for PubMed contains the keywords "Pediatric," "Etiology," and "Urinary Tract Infections," among numerous others. We will concentrate on English-language studies published between 2013 and 2023, which mainly targeted children between zero and 18. Non-full-text publications, studies without clear evidence, and articles without peer review are excluded. We will use the Preferred Reporting Items for Systematic Reviews and Meta-Analyses (PRISMA) criteria to evaluate the quality after obtaining pertinent data. The collected data will be subjected to narrative synthesis or meta-analysis, depending on how uniform the research is. While recent English papers are given priority in our search, we recognize that these limitations may result in the exclusion of pertinent studies. However, this approach will guarantee an annual update with the most recent research, assisting medical practitioners in providing the best care for kids with UTIs. The PRISMA flow diagram is presented in Figure 1.

Aetiology of UTIs in children

Children's UTIs are primarily caused by bacteria, with Escherichia coli being the most prevalent pathogen [11]. However, other species, including Klebsiella, Proteus, Enterococcus, and Staphylococcus saprophyticus, can also contribute to UTIs [12]. In certain clinical scenarios, Candida species may lead to fungal UTIs, while viral cystitis can be caused by viruses like adenovirus and herpes simplex, albeit less commonly than bacterial infections [13]. Parasitic UTIs are rare in developed countries but may occur in regions where specific parasites are endemic [14]. Hematogenous spread, particularly from fungal and staphylococcal infections, is more common in individuals who are ill, blocked, or immunocompromised [11]. Escherichia coli, with its various virulence factors, remains the most common cause of UTIs in children and adults [12]. Recognizing the diverse aetiology of UTIs is crucial for accurate diagnosis, appropriate treatment, and prevention strategies, especially in children with recurrent infections or underlying risk factors. Tailored approaches to managing this condition in pediatric populations are essential [11,14]. The aetiology and epidemiology of UTIs in children are detailed in Table 1.

**Table 1:** Aetiology and epidemiology of UTIs in children [12-14] UTIs: Urinary tract infections

Primary Cause	Bacteria entering the urinary tract
Common pathogens	Escherichia coli
Other pathogens	Klebsiella, Proteus, Enterococcus, Staphylococcus saprophyticus
Exceptional cases (hematogenous spread)	It occurs mainly in debilitated, obstructed, or immunocompromised patients, commonly due to fungal and staphylococcal infections.
Viral causes	Adenovirus, herpes simplex (less common)
Fungal causes	Candida species (typically associated with yeast infections)
Parasitic UTIs	Rare in developed countries, prevalent where certain parasites are endemic
Importance of understanding	Helps in accurate diagnosis, treatment, and prevention, especially in recurrent cases or those with underlying risk factors.

Treatment strategy for UTIs in children

UTIs are a significant concern in children, necessitating empirical antibiotic treatment while awaiting culture results [15]. Common first-line oral antibiotics for uncomplicated cases include amoxicillin-clavulanate, cefixime, or trimethoprim-sulfamethoxazole (TMP-SMX) [16]. In severe cases or when resistance is suspected, broader-spectrum antibiotics may be considered initially [16]. Treatment duration varies, with three to seven days for uncomplicated cases and longer courses for upper urinary tract involvement [15]. Research suggests treating acute pyelonephritis (APN) in children for six to nine days is as effective as longer courses [17]. Parenteral antibiotic therapy for ≤seven days may be suitable for bacteremic UTIs in infants <60 days [18]. Close monitoring and follow-up urine cultures are crucial [18]. Preventing recurrent UTIs involves minimizing constipation, promoting good hygiene, and encouraging healthy habits [1,2]. A well-rounded diet, including probiotics from yoghurt, hydration, and frequent toilet breaks, supports urinary tract health [4,10,15]. Other preventive measures include cotton underwear, proper wiping techniques, and exercise [17,19].

Antibiotic resistance in UTIs, especially against Escherichia coli, poses a significant challenge [20]. Prudent antibiotic use, accurate diagnoses through urine cultures, and international antimicrobial stewardship programs are essential [17]. Preventive measures, such as cleanliness and addressing constipation, can reduce the need for antibiotics [19,21]. International collaboration and ongoing research are vital to understanding and combating antibiotic resistance [8]. Tailored treatments based on culture and sensitivity results are crucial, with alternative antibiotics like ceftriaxone, ciprofloxacin, or nitrofurantoin considered in cases of resistance [22]. Preventive strategies, including hygiene practices and prophylaxis, are key in mitigating the impact of antibiotic resistance [23]. In summary, a rational, individualized approach to antibiotic selection, guided by local resistance patterns, is crucial in managing pediatric UTIs to curb antibiotic resistance development and spread [3]. Understanding UTIs in children is critical for parents, caregivers, and healthcare professionals, emphasizing the importance of preventive measures and responsible antibiotic use [24,25]. Antibiotic resistance and UTIs in children are detailed in Table 2.

**Table 2:** Antibiotic resistance and UTIs in children [20-25] UTIs: Urinary tract infections

Antibiotic resistance concern	Significant in both children and adults
Rise in resistance	Noted in common uropathogens, especially Escherichia coli, against commonly used antibiotics [20]
Impact on treatment selection	Influences the choice of empirical antibiotics, sometimes leading to broader-spectrum agents [21]
Tailored treatment	Based on culture and sensitivity results
Alternative antibiotics for resistance	Include second-line agents like ceftriaxone, ciprofloxacin (in older children), and nitrofurantoin [22]
Prevention strategies	Maintaining good hygiene and prophylactic measures in high-risk cases [23]
Importance of addressing resistance	Vital for patient care and public health. Necessary to retain drug efficacy for future generations [24]
General UTIs concerns	Common across all ages but with varying impacts. Critical for parents, caregivers, and healthcare professionals to understand UTIs in children for proper diagnosis [25]

Diagnostic approaches in pediatric UTIs

UTIs in children arise from bacteria entering and multiplying in the urinary tract, with girls being more susceptible due to a shorter urethra. Symptoms vary with age, appearing non-specific in babies and young children and becoming more typical (pain, frequency) as they grow. Risk factors encompass vesicoureteral reflux, constipation, poor hygiene, infrequent urination, structural abnormalities, and family history [26,27]. Personalized imaging is essential; ultrasound identifies structural issues [22], contrast-enhanced voiding cystourethrogram (VCUG) assesses bladder and urethra [25], CT scans provide high-resolution images [24], and MRI offers radiation-free detailed imaging [23]. Nuclear medicine scans evaluate kidney function [27]. Decision-making relies on clinical evaluations with adjustments based on responses [28]. Urine samples contribute to diagnosis; a catheter may be required for newborns [28]. Urinalysis and culture confirm pathogens [29]. Prompt detection is crucial to prevent kidney infections. Tailored antibiotics are essential, and completing the course is necessary to prevent recurrence and antibiotic resistance [29]. Preventive measures involve promoting hygiene, ensuring complete bladder emptying, treating constipation, and addressing urinary abnormalities [10]. For recurrent UTIs, low-dose antibiotic therapy may be recommended [26,30]. A summary table of the included studies is provided in Table 3.

**Table 3:** A summary table of the included studies

Author(s)	Year	Outcome/conclusion
Leung et al. [1]	2019	Investigated urinary tract infections in children, emphasizing recent patents, inflammation, and drug discovery in the field.
Korbel et al. [2]	2017	Explored the clinical aspects of diagnosing and managing urinary tract infections in the pediatric population.
Simões et al. [3]	2020	Provided a comprehensive overview of urinary tract infections in paediatrics, offering valuable insights into the subject.
Flores et al. [4]	2015	Reviewed the epidemiology, mechanisms, and various treatment options for urinary tract infections.
Ma et al. [5]	2004	Examined the aetiology and epidemiology of urinary tract infections in the pediatric population.
Larcombe et al. [6]	2015	Explored the recurrence of urinary tract infections in children, offering insights into the clinical evidence surrounding the topic.
Czajkowski et al. [7]	2021	Investigated urinary tract infections in women, specifically focusing on the menopausal population.
Pokrajac et al. [8]	2018	Examined the influence of plasminogen activator inhibitor-1 gene polymorphism on renal scarring after infants' first febrile urinary tract infection.
Godaly et al. [9]	2015	Explored innate immunity and genetic determinants influencing susceptibility to urinary tract infections.
Jung et al. [10]	2019	Discussed the aetiology and management of recurrent urinary tract infections in postmenopausal women.
Baraboutis et al. [11]	2010	Explored primary Staphylococcus aureus urinary tract infections and the role of undetected hematogenous seeding of the urinary tract.
McLellan et al. [12]	2016	Explored the pathogenesis and outlook of urinary tract infections, providing insights into the molecular trends in medicine.
Flores et al. [13]	2019	Discussed the pathophysiology, treatment, and prevention of catheter-associated urinary tract infections in the context of spinal cord injuries.
National Collaborating Centre for Women's' and Children's' Health (UK) [14]	2007	Published guidelines on diagnosing, treating, and managing urinary tract infections in children.
Hudson et al. [15]	2022	Examined complementary medicine for treating urinary tract infections among pregnant women and children, presenting findings on the efficacy of alternative approaches.
Fitzgerald et al. [16]	2012	Analyzed the use of antibiotics for treating lower urinary tract infections in children through a systematic review, contributing to evidence-based practices.
Fox et al. [17]	2020	Investigated the comparative effectiveness of antibiotic treatment duration in children with pyelonephritis, providing insights into optimal treatment durations.
Desai et al. [18]	2019	Explored the duration of parenteral antibiotic therapy in young infants with bacteremic urinary tract infections, contributing to pediatric treatment protocols.
Fasugba et al. [19]	2020	Conducted a systematic review on increased fluid intake for preventing urinary tract infections in adults and children in various settings, providing valuable insights for infection prevention strategies.
Mathew [20]	2010	Presented a systematic review of randomized controlled trials on antibiotic prophylaxis following urinary tract infection in children, contributing to evidence-based practices in pediatric care.
White [21]	2011	Discussed the diagnosis and treatment of urinary tract infections in children, offering clinical insights for healthcare practitioners.
Ahmed et al. [22]	1998	Provided an evaluation and treatment approach for urinary tract infections in children, serving as a reference for clinical practice.
Pietrucha et al. [23]	2016	Explored the diagnosis, treatment, and prevention of urinary tract infections, contributing to understanding microbial spectrums and effective interventions.
Chenoweth et al. [24]	2014	Focused on diagnosing, managing, and preventing catheter-associated urinary tract infections, providing insights into best practices for healthcare settings.
Harambat et al. [25]	2012	Investigated the epidemiology of chronic kidney disease in children, contributing to understanding this condition in pediatric populations.
Uwaezuoke [26]	2016	Conducted a narrative review on the prevalence of urinary tract infection in children with severe acute malnutrition, offering insights into the association between malnutrition and urinary tract health.
Ross et al. [27]	1999	Explored pediatric urinary tract infections and reflux, providing insights into the relationship between these conditions, which is valuable for family physicians.
Mori et al. [28]	2007	Summarized the National Institute for Health and Clinical Excellence (NICE) guidance on diagnosing and managing urinary tract infections in children, offering a concise reference for healthcare practitioners.
Autore et al. [29]	2023	Provided guidelines and recommendations for antibiotic prophylaxis in preventing urinary tract infections in children, contributing to evidence-based practices from the Emilia-Romagna Pediatric Urinary Tract Infections Study Group.
Becknell et al. [30]	2015	Examined the diagnosis, evaluation, and treatment of acute and recurrent pediatric urinary tract infections, offering expertise in anti-infective therapy and contributing to best practices.

## Conclusions

In conclusion, this meta-analysis underscores crucial practice standards for managing pediatric UTIs, synthesizing evidence-based insights to guide clinical decision-making. Establishing evidence-based protocols emerges as a cornerstone, focusing on incorporating high-quality studies identified through systematic reviews and meta-analyses. Standardized protocols enhance consistency in diagnosis and treatment, ensuring that clinical practices are firmly grounded in the robust understanding derived from the literature. Optimizing antibiotic selection and duration represents a pivotal outcome of this meta-analysis. Tailoring treatment plans based on local resistance patterns becomes imperative to achieve optimal clinical outcomes and mitigate the looming threat of antibiotic resistance. Age-stratified management guidelines offer a nuanced approach, recognizing the diverse clinical presentations and treatment responses across pediatric age groups.

As informed by aggregated evidence on non-antibiotic interventions, preventive strategies emphasize lifestyle modifications, increased fluid intake, and probiotic use. These evidence-based measures, disseminated to healthcare professionals, parents, and caregivers, aim to curtail the incidence of recurrent UTIs and promote overall urinary tract health. Enhanced imaging guidelines, derived from insights into diagnostic accuracy and utility, advocate for personalized and cost-effective approaches, minimizing unnecessary procedures. The meta-analysis encourages a culture of continuous quality improvement, urging healthcare institutions to stay abreast of emerging evidence and actively participate in collaborative initiatives. Ultimately, patient and public education emerge as pivotal components, translating synthesized evidence into accessible materials for informed decision-making and treatment plan adherence. By embracing these practice standards, healthcare professionals can enhance the precision and efficiency of pediatric UTI management, ensuring alignment with the most robust and current evidence available.
